# Correlation of Total Polyphenol and Flavonoid Content With Antioxidant Activity of Caucasian Bee Honey

**DOI:** 10.1002/fsn3.4611

**Published:** 2025-01-09

**Authors:** Tuğba Nigar Bozkuş

**Affiliations:** ^1^ Laboratory Technology Program Artvin Vocational School, Artvin Çoruh University Artvin Türkiye

**Keywords:** antioxidant activity, Caucasian bee, flavonoid content, honey, polyphenol content

## Abstract

Honey is a natural product gathered by honeybees from the pollen and nectar of various plants and flowers. The homeland of the Caucasian honey bee, which draws attention with its honey production and is one of the most productive bee races known in the world, is Northeastern Anatolia in Türkiye. This study aims to determine and correlate the phenolic content and antioxidant activity of 54 honey samples obtained from the most important gene centers of the Caucasian bee in Türkiye. Total polyphenol and flavonoid contents and ferric reducing antioxidant power of aqueous honey extracts were in the range of 9.95–66.34 mg GA/100 g, 0.76–14.24 mg Q/100 g, 63.00–150.00 mg T/100 g, respectively. All analysis methods of Caucasian bee honey samples showed statistically significant differences according to location. The variation in phenolic content and antioxidant activities in Caucasian bee honey is attributed to the region's specific geographical location and vegetation. Furthermore, a robust and significant correlation was identified between the antioxidant activity of the honey samples and the total levels of polyphenols and flavonoids. Thanks to these features, Caucasian bee honey can be an important nutritional and commercial product.

## Graphical abstract

This study includes the correlation between the phenolic contents and antioxidant activities of 54 Caucasian bee honey samples obtained from the Artvin and Ardahan regions, the most important gene centers of the Caucasian bee in Türkiye. The difference in the phenolic content and antioxidant activities of Caucasian bee honey is due to its geographical location and vegetation, and a strong and significant correlation was found between these parameters. Thanks to these features, Caucasian bee honey can be an important nutritional and commercial product.

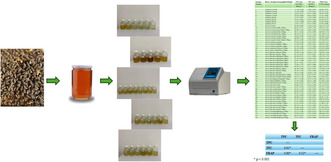

## 1 Introduction

Honey is a bee product widely consumed worldwide for health protection and treatment purposes as well as nutrition, and is a natural substance collected by bees from the pollen and nectar of plants and flowers (Kropf et al. 2010; Akgün, Çelik, and Kelebekli 2021). The chemical composition of honey varies based on several factors, including its geographical and botanical origin, the season of collection, the bee race, and the method of storage (Kaskoniene and Venskutonis 2010). Honey has biological properties such as antioxidant, antibacterial (Otmani et al. 2021), antimicrobial (Vica et al. 2021), antiviral (Watanabe et al. 2014), anti‐inflammatory (Cianciosi et al. 2021), antiulcer (Vandamme et al. 2013), and antitumoral (Jaganathan et al. 2011) activity.

Caucasian honey bee (*Apis mellifera Caucasia*) race, one of the most productive bee races defined in the world, is one of the most important honey bee genotypes in Türkiye and its homeland is Northeastern Anatolia (Akyol et al. 2014; Gül and Nergiz, 2022). The Caucasian honey bee, which is adapted to temperate climates and high‐altitude areas, is notable for its long tongue, extensive use of propolis, low tendency to swarm, and honey production capabilities (Uçak Koç and Karacaoğlu 2011; Akyol et al. 2014). Artvin and Ardahan are the most important gene centers of the Caucasian bee in Türkiye and are isolated regions closed to foreign beekeepers (Gül and Nergiz 2022).

It has been determined that there are limited studies in the literature on bee products obtained from the Caucasian bee (Silici and Kutluca 2005; Şahinler and Kaftanoğlu 2005; Saral et al. 2019; Kekeçoğlu et al. 2020). However, none of these studies have focused so much on the phenolic content and antioxidant activity of Caucasian bee honey samples. For this reason, this study includes 54 Caucasian bee honey samples obtained from the Artvin and Ardahan regions, the most important gene centers of the Caucasian bee in Türkiye, and is the first research in its field. As with all bee products, the choice of extraction solvent plays a crucial role in determining the phenolic content and antioxidant activity of honey. For this reason, in this study, water, which is the most natural solvent, was preferred to extract honey, which is a natural product. This study seeks to assess the total polyphenol and flavonoid contents, as well as the antioxidant activities, of aqueous extracts from honey samples collected from Caucasian bees, and to investigate the relationship between these parameters.

This research may provide valuable insights into the phenolic content and antioxidant activity of honey from the Caucasian honey bee, a species renowned for its high honey production. By analyzing 54 honey samples from key genetic centers in Northeastern Anatolia, this study may reveal significant regional variations in polyphenol and flavonoid content, as well as antioxidant activity. This is particularly noteworthy because it underscores the impact of geographical and botanical diversity on honey's nutritional and commercial value. The robust correlation identified between antioxidant activity and polyphenol/flavonoid levels may support the potential health benefits and marketability of Caucasian bee honey. This research could enhance the ability to standardize honey quality and optimize its health benefits.

## 2 Materials and Methods

### 2.1 Honey Extracts

A total of 54 natural honey samples were obtained from Artvin (*N* = 46) and Ardahan (*N* = 8), through the Artvin Beekeepers Association. These samples were collected from beekeepers in fixed regions between Artvin and Ardahan, who did not practice migratory beekeeping. Fifteen grams of each honey sample was weighed. Then, 25 mL of pure water was added. Honey samples were vortexed and incubated in a shaking incubator at 30°C and 150 rpm for 24 h. At the end of the incubation, honey extracts were centrifuged at 2057 *g* for 10 min and filtered with filter paper. Thus, 600 mg/mL aqueous stock Caucasian bee honey extracts were prepared. Analyses were repeated four times for each sample (*n* = 4).

### 2.2 Total Polyphenol Content (TPC)

The modified Folin–Ciocalteu method (Horzic et al. 2009) was used. Honey extract (12.5 μL), 1:10 diluted Folin–Ciocalteu reagent (62.5 μL), and 20% sodium carbonate solution (125 μL) were added to 96‐well microplates and incubated for 30 min in the dark at room temperature. Absorbance measurement was performed at 700 nm in a microplate reader. Results were converted to mg Gallic acid (GA)/100 g honey using the gallic acid standard graphic.

### 2.3 Total Flavonoid Content (TFC)

The Aluminum chloride colorimetric method (Chang et al. 2002) was used. Honey extract (20 μL), 80% ethanol (172 μL), 10% aluminum chloride (4 μL), and 1 M potassium acetate solution (4 μL) were added to 96‐well microplates and incubated at room temperature in the dark for 40 min. Absorbance measurement was performed at 415 nm in a microplate reader. Results were converted to mg Quercetin (Q)/100 g honey using the quercetin standard graphic.

### 2.4 Ferric (Fe^3+^) Reducing Antioxidant Power (FRAP)

The modified ferricyanide method (Mohtar et al. 2020) was used. Honey extract (40 μL), 0.2 M phosphate buffer (pH: 6.6) (100 μL) and potassium hexacyanoferrate (100 μL) were added and incubated at 50°C and in the dark for 20 min. After incubation, the samples were cooled. 10% trichloroacetic acid (100 μL) was added to the mixture and this mixture was centrifuged at 3000 g for 10 min. The upper phases of the centrifuged samples (100 μL) were taken into a 96‐well microplate, and distilled water (100 μL) and iron (III) chloride (20 μL) were added to them. The final mixture was incubated at room temperature in the dark for 5 min. Absorbance measurement was performed at 700 nm in a microplate reader. Results were converted to mg Trolox (T)/100 g honey using trolox standard graphic.

### 2.5 Statistics

Data were evaluated statistically using the R Program (Version 4.3). To reveal the relationship between the groups, normality analysis was performed, and it was seen that the data were normally distributed only in the FRAP analysis results. The independent samples *t*‐test, Mann–Whitney *U* test, and Pearson correlation were employed.

## 3 Results

Aqueous extracts of Caucasian bee honey samples were prepared and total polyphenol contents (TPC), total flavonoid contents (TFC), and ferric (Fe^3+^) reducing antioxidant power amounts (FRAP) were analyzed. The results are given in Table 1 as mg GA/100 g honey, mg Q/100 g honey, and mg T/100 g honey, respectively.

**TABLE 1:** Analyses results of aqueous extracts of Caucasian bee honey samples (Arithmetic mean ± SD, *n* = 4).

Sample number	Honey samples (Geographical origin)	TPC (mg GA/100 g honey)	TFC (mg Q/100 g honey)	FRAP (mg T/100 g honey)
1	Ardahan‐Central	11.18 ± 0.69	1.00 ± 0.01	64.00 ± 0.37
2	Ardahan‐Central	17.33 ± 0.84	1.78 ± 0.16	92.75 ± 1.35
3	Ardahan‐Central	12.28 ± 0.15	0.91 ± 0.08	70.50 ± 0.58
4	Ardahan‐Central	10.20 ± 0.48	0.66 ± 0.02	73.25 ± 0.41
5	Ardahan‐Hanak	13.35 ± 0.67	1.36 ± 0.08	88.30 ± 1.10
6	Ardahan‐Hanak	10.67 ± 0.28	0.76 ± 0.05	63.00 ± 0.71
7	Ardahan‐Hanak	11.97 ± 0.94	0.77 ± 0.08	73.05 ± 0.50
8	Ardahan‐Posof	9.95 ± 1.07	0.97 ± 0.03	65.20 ± 0.49
9	Artvin‐Central	22.08 ± 0.82	1.84 ± 0.10	124.13 ± 1.28
10	Artvin‐Central‐Ağıllar Village	28.67 ± 2.51	2.94 ± 1.05	120.53 ± 1.81
11	Artvin‐Central‐Aşağı Maden Village	28.00 ± 1.50	2.06 ± 0.45	125.38 ± 0.82
12	Artvin‐Central‐Erenler Village	30.17 ± 2.62	2.75 ± 0.99	128.89 ± 1.72
13	Artvin‐Central‐Erenler Village	20.14 ± 1.78	2.71 ± 1.36	121.54 ± 1.19
14	Artvin‐Central‐Yanıklı Village	30.68 ± 0.96	2.54 ± 0.95	129.42 ± 1.50
15	Artvin‐Central‐Yanıklı Village	26.04 ± 1.33	1.92 ± 0.15	126.83 ± 0.85
16	Artvin‐Central‐Yukarı Maden Village	34.18 ± 2.09	2.74 ± 0.03	133.80 ± 0.51
17	Artvin‐Ardanuç‐Central	31.63 ± 1.45	1.62 ± 0.27	125.87 ± 1.91
18	Artvin‐Ardanuç‐Bağlıca Village	19.70 ± 0.76	5.60 ± 0.91	108.80 ± 2.41
19	Artvin‐Ardanuç‐Çakıllar Village	21.98 ± 1.42	1.57 ± 0.29	103.56 ± 2.06
20	Artvin‐Ardanuç‐Zekeriya Village	22.18 ± 0.90	1.38 ± 0.07	103.03 ± 2.04
21	Artvin‐Arhavi‐Şahinkaya Village	24.56 ± 1.33	3.81 ± 1.14	124.28 ± 0.73
22	Artvin‐Borçka‐Akpınar Village	23.20 ± 1.74	2.49 ± 1.14	120.45 ± 1.24
23	Artvin‐Borçka‐Avcılar Village	47.15 ± 2.87	6.72 ± 0.21	140.19 ± 1.59
24	Artvin‐Borçka‐Çifteköprü Village	45.88 ± 2.15	5.11 ± 0.98	141.39 ± 1.79
25	Artvin‐Borçka‐Çifteköprü Village	27.12 ± 2.52	3.54 ± 0.23	134.81 ± 1.03
26	Artvin‐Borçka‐Çifteköprü Village	32.87 ± 1.44	3.82 ± 1.10	134.28 ± 1.60
27	Artvin‐Borçka‐Çifteköprü Village	29.31 ± 0.81	3.32 ± 0.27	133.37 ± 1.65
28	Artvin‐Borçka‐Demirciler Village	35.86 ± 1.27	4.69 ± 0.46	134.95 ± 2.29
29	Artvin‐Borçka‐Demirciler Village	43.26 ± 1.18	5.31 ± 0.93	140.10 ± 2.12
30	Artvin‐Borçka‐Düzköy Village	66.34 ± 2.96	9.95 ± 0.76	150.00 ± 2.74
31	Artvin‐Borçka‐Güneşli Village	38.16 ± 1.88	5.60 ± 0.86	131.06 ± 1.41
32	Artvin‐Borçka‐Macahel (Camili)	24.16 ± 2.48	14.24 ± 1.28	120.19 ± 2.42
33	Artvin‐Borçka‐Macahel (Camili)‐Efeler Village	19.25 ± 1.04	4.89 ± 0.37	115.27 ± 0.42
34	Artvin‐Borçka‐Macahel (Camili)‐Maral Village	22.55 ± 1.54	6.06 ± 0.43	135.00 ± 2.81
35	Artvin‐Kemalpaşa‐Köprücü Village	58.77 ± 3.04	12.27 ± 1.68	140.05 ± 1.93
36	Artvin‐Murgul‐Başköy Village	31.93 ± 2.43	4.33 ± 1.48	121.06 ± 1.20
37	Artvin‐Murgul‐Çimenli Village	36.05 ± 0.76	4.17 ± 0.39	126.97 ± 0.92
38	Artvin‐Şavşat‐Central	29.40 ± 2.37	4.12 ± 0.99	131.59 ± 0.53
39	Artvin‐Şavşat‐Çayağzı Village	22.81 ± 1.88	1.99 ± 0.23	125.91 ± 1.28
40	Artvin‐Şavşat‐Karaağaç Village	25.21 ± 1.34	1.80 ± 0.29	123.80 ± 1.52
41	Artvin‐Şavşat‐Kocabey Village	26.88 ± 0.25	2.28 ± 0.26	125.67 ± 1.55
42	Artvin‐Şavşat‐Maden Village	20.21 ± 1.72	1.63 ± 0.11	121.06 ± 1.14
43	Artvin‐Şavşat‐Meydancık‐Tepebaşı Village	19.63 ± 0.88	9.30 ± 2.06	107.13 ± 2.19
44	Artvin‐Şavşat‐Tepeköy Village	20.49 ± 1.67	1.45 ± 0.21	90.87 ± 1.37
45	Artvin‐Şavşat‐Veliköy Village	25.91 ± 0.93	1.58 ± 0.12	104.38 ± 0.82
46	Artvin‐Şavşat‐Yamaçlı Village	21.87 ± 0.89	1.92 ± 0.41	110.24 ± 1.64
47	Artvin‐Şavşat‐Yavuzköy Village	21.65 ± 0.70	1.56 ± 0.18	97.40 ± 1.18
48	Artvin‐Şavşat‐Yavuzköy Village	20.76 ± 1.41	1.55 ± 0.09	89.13 ± 0.71
49	Artvin‐Şavşat‐Yoncalı Village	29.68 ± 2.10	3.51 ± 0.87	117.69 ± 0.92
50	Artvin‐Şavşat‐Yoncalı Village	25.26 ± 0.43	1.87 ± 0.13	101.97 ± 0.10
51	Artvin‐Yusufeli‐Bakırtepe Village	16.15 ± 1.06	1.09 ± 0.19	100.72 ± 1.06
52	Artvin‐Yusufeli‐Boyalı Village	39.29 ± 2.19	3.97 ± 0.50	140.64 ± 1.96
53	Artvin‐Yusufeli‐Erenköy Village	24.35 ± 2.13	1.78 ± 0.18	117.26 ± 2.53
54	Artvin‐Yusufeli‐Kılıçkaya Village	15.31 ± 0.21	0.96 ± 0.06	102.36 ± 0.18

Table 2 Presents the mean, median, standard deviation (SD), and standard error (SE) values for the Artvin (*N* = 46) and Ardahan (*N* = 8) honey groups, as determined by the analysis methods. It is seen that the average of the Artvin group is higher in all analysis methods.

**TABLE 2:** Group descriptives by analysis methods.

Method	Group	*N*	Mean	Median	SD	SE
TPC	Ardahan	8	12.12	11.57	2.39	0.85
	Artvin	46	28.84	25.98	10.38	1.53
TFC	Ardahan	8	1.03	0.94	0.37	0.13
	Artvin	46	3.75	2.75	2.87	0.42
FRAP	Ardahan	8	73.76	71.78	11.13	3.94
	Artvin	46	121.80	124.20	14.40	2.12

According to the Shapiro–Wilk, Kolmogorov–Smirnov, and Anderson‐Darling tests of the normality assumption, which were examined before examining the statistical significance of the differences, it can be said that only the data of the FRAP analysis were normally distributed (*p* > 0.05). When the homogeneous distribution of variances, which is a prerequisite for the *t*‐test for FRAP analysis, was examined with Levene's test, it was seen that the variances were distributed homogeneously (*p* > 0.05).

According to all analysis methods, the differences between the locations were found to be statistically significant (*p* < 0.001, df = degrees of freedom) (Table 3).

**TABLE 3:** Mann–Whitney *U* (*U*) and *t*‐test (*T*) results.

Method	Test	Statistics	df	*p*
TPC	*U*	2.00	52	< 0.001
TFC	*U*	14.50	52	< 0.001
FRAP	*T*	8.95	52	< 0.001

Pearson correlation coefficient (*r*) was used to correlate the antioxidant activities (FRAP) of the extracts with their polyphenol (TPC) and flavonoid (TFC) contents (Table 4).

**TABLE 4:** Correlation matrix showing the interrelations among TPC, TFC and FRAP.

	TPC	TFC	FRAP
TPC	—		
TFC	0.61*	—	
FRAP	0.80*	0.52*	—

* *p* < 0.001.

All correlations were statistically significant, and the highest correlation was found between TPC and FRAP (*r* = 0.80). This shows that polyphenols and flavonoids contribute to the antioxidant activity of honey. These data showed that the antioxidant activities of honey samples were significantly related to polyphenol and flavonoid content amounts of honey samples.

## 4 Discussion

In this study, total polyphenol content of aqueous honey extracts was determined in the range of 9.95 ± 1.07–66.34 ± 2.96 mg GA/100 g honey and total flavonoid content was determined in the range of 0.76 ± 0.05–14.24 ± 1.28 mg Q/100 g honey, while ferric (Fe^3+^) reducing antioxidant power amount was determined in the range of 63.00 ± 0.71–150.00 ± 2.74 mg T/100 g honey. According to the results, Caucasian bee honey samples were found to be statistically significant in terms of location in all analysis methods. A strong correlation was observed between the phenolic content of honey and its antioxidant activities.

Türkiye is home to various honey bee races and ecotypes, but pure stocks are often hybridized due to migratory beekeeping practices and commercial queen bee breeding (Akyol and Kaftanoğlu 2001). Caucasian honey bee is housed in an isolated area close to the entrance of different species of bees in the Artvin and Ardahan regions, which are only open to each other in Türkiye, and thus its gene is protected (Gül and Nergiz 2022). For this reason, in this study, honey was obtained from Caucasian bees located in fixed regions between Artvin and Ardahan, where migratory beekeeping was not carried out.

Two hundred and sixty honey plant species have been identified in Artvin, which has a mountainous and rugged terrain with forests (Eminağaoğlu 2015). In Ardahan province, steppe vegetation is dominant, there is no forest area, and another important vegetation part is Alpine grassland (Şık et al. 2017). For this reason, while most of the honey produced in Artvin is a flower, chestnut, linden, rhododendron, and astragalus honey, the honey produced in Ardahan is a flower, clover, astragalus, and thyme honey (Türkiye Cumhuriyeti Tarım ve Ormancılık Bakanlığı 2023). Since chestnut trees are widespread in the Borçka and Murgul districts of Artvin province and chestnut honey is specially produced in these regions, this honey was provided as chestnut honey. In addition, the honey provided from Kemalpaşa district of Artvin is chestnut honey. In addition, since flower honey is mostly produced in other districts of Artvin, flower honey samples were provided for use in the study. The honey supplied from the Ardahan region is the most produced flower honey depending on the vegetation.

Among Caucasian bee honey samples, it was determined that Artvin‐Borçka‐Düzköy Village honey sample had the highest polyphenol content (66.34 ± 2.96 mg GA/100 g honey) and antioxidant activity (150.00 ± 2.74 T/100 g honey) and Artvin‐Borçka‐Macahel honey sample had the highest flavonoid content (14.24 ± 1.28 Q/100 g honey). The reason for these high results is that both honey samples contain chestnut components, which are rich in botanical resources due to the abundant chestnut trees in that region. In various studies conducted with different types of honey, it has been reported that dark‐colored honey samples, especially chestnut honey, show higher antioxidant values depending on the number of phenolic substances and have higher activity than other kinds of honey (Al‐Mamary, Al‐Meeri, and Al‐Habori 2002; Bertoncelj et al. 2007; Küçük et al. 2007; Tezcan et al. 2011). Therefore, significant differences in the amounts of total polyphenols and flavonoids between the analyzed honey samples are due to differences in floral sources (Pichichero, Canuti, and Canini 2009). In addition, the Ardahan‐Hanak honey sample had the lowest total phenolic content and antioxidant activity values. It can be said that the main difference between these two regions in terms of phenolic content and antioxidant activity comes from the geographical location and vegetation. Thanks to these properties, Caucasian bee honey can be a nutritional and commercially important product.

Since honey is a food product consumed in its most natural form, the most appropriate and most natural solvent should be used when preparing the extract. For this reason, as in most studies, water was used as a natural solvent in this study (Nagai et al. 2001; Beretta et al. 2005; Bertoncelj et al. 2007; Al et al. 2009; Pichichero, Canuti, and Canini 2009; Velimirovic et al. 2023; Bozkuş 2024).

Al et al. (2009) in the study conducted with aqueous extracts of 24 different honey samples in Romania, total polyphenol amount of the honey samples was determined to be between 2.00–125.00 mg GA/100 g, and total flavonoid content amount was determined to be between 0.91 and 28.25 mg Q/100 g. In addition, it has been reported that the highest amount of flavonoids is in honey with multiple flora (Al et al. 2009). Socha et al. (2009) determined total polyphenol amount of aqueous extract of 10 different honey samples as 21.7–75.3 mg Q/100 g GA, and total flavonoid amount as 6.9–28.5 mg Q/100 g honey. It appears that the findings of the studies presented here are higher than the total phenolic substance findings of this study. It is thought that the main reason for this is due to bee race, geographical location and vegetation of Artvin and Ardahan regions. Furthermore, study results revealed that honey samples with high polyphenol content also have elevated levels of flavonoids (Al et al. 2009; Socha et al. 2009). In this respect, the results obtained in both studies and the results of this study are consistent with each other. In addition, in a study conducted by Saral et al. (2019) and comparing *Apis mellifera Caucasia*, *Apis mellifera Anatoliaca*, *Apis mellifera Syriaca*, and *Apis mellifera Carnica* bee races, it was concluded that vegetation is a more effective factor than bee race in terms of antioxidant activity, and the study supports this study.

The findings of this study align with those of numerous researchers, revealing a strong correlation between antioxidant activities and total polyphenol and flavonoid content (Blasa et al. 2006; Alvarez‐Suarez et al. 2010; Islam et al. 2012; Alves et al. 2013). Consequently, this study further supports the thesis that phenolic content significantly contributes to the antioxidant activity of honey. Additionally, it reinforces the commonly accepted notion that honey's antioxidant activity varies considerably based on its floral and botanical sources, which are influenced by location. Based on these results, Caucasian bee honey can be considered a rich source of natural polyphenols and flavonoids, offering high nutritional and therapeutic value along with its antioxidant properties.

## 5 Conclusions

The polyphenol and flavonoid contents and antioxidant activities of 54 different honey samples obtained from the regions that are the gene centers of the Caucasian bee were determined in detail for the first time in this study. The results showed that Caucasian bee honey has varying degrees of strong antioxidant potential and high polyphenol and flavonoid content levels. The high correlation between phenolic content amounts and antioxidant activity revealed that these polyphenols and flavonoids are the major components responsible for the antioxidant effect of honey. In light of these data, Caucasian bee honey can be considered a natural source of antioxidants. In addition, it is understood that the most important factor determining the phenolic content amounts and antioxidant activities of Caucasian bee honey is the geographical location and plant diversity of the region where it is produced.

Although honey obtained from Caucasian bees was preferred in this study, it is possible to say that various types of studies can be carried out with honey obtained from different bee races. In addition, biologically valuable components such as polyphenols and flavonoids found in Caucasian bee honey can be examined in detail with future quantitative analysis studies. Investigating the health effects of Caucasian bee honey on experimental animals may benefit the use of honey as a high value‐added product in the health sector. The biochemical properties obtained in this study may be reinforced with the physicochemical and microbiological analyses in future studies to fully determine the quality of the honey produced and used in the region.

## Author Contributions

**Tuğba Nigar Bozkuş:** conceptualization (lead), formal analysis (lead), funding acquisition (lead), investigation (lead), methodology (lead), project administration (lead), supervision (lead), writing – original draft (lead), writing – review and editing (lead).

## Conflicts of Interest

The author declares no conflicts of interest.

## Data Availability

The data are available from the corresponding author upon reasonable request.
